# Comprehensive development and testing of the ASIST-GBV, a screening tool for responding to gender-based violence among women in humanitarian settings

**DOI:** 10.1186/s13031-016-0071-z

**Published:** 2016-04-20

**Authors:** A. L. Wirtz, N. Glass, K. Pham, N. Perrin, L. S. Rubenstein, S. Singh, A. Vu

**Affiliations:** Center for Public Health and Human Rights, Department of Epidemiology, Johns Hopkins School of Public Health, Baltimore, USA; Johns Hopkins School of Nursing, Baltimore, USA; Department of Emergency Medicine, Johns Hopkins Medical Institute, Baltimore, USA; Department of International Health, Johns Hopkins School of Public Health, Baltimore, USA; Center for Health Research, Kaiser Permanente Northwest, Portland, USA; Department of Internal Medicine, Johns Hopkins Medical Institute, Baltimore, USA

**Keywords:** Gender-based violence, Screening, Conflict, Refugees, Internally-displaced person, Humanitarian setting, Ethiopia, Colombia

## Abstract

**Background:**

Conflict affected refugees and internally displaced persons (IDPs) are at increased vulnerability to gender-based violence (GBV). Health, psychosocial, and protection services have been implemented in humanitarian settings, but GBV remains under-reported and available services under-utilized. To improve access to existing GBV services and facilitate reporting, the ASIST-GBV screening tool was developed and tested for use in humanitarian settings. This process was completed in four phases: 1) systematic literature review, 2) qualitative research that included individual interviews and focus groups with GBV survivors and service providers, respectively, 3) pilot testing of the developed screening tool, and 4) 3-month implementation testing of the screening tool. Research was conducted among female refugees, aged ≥15 years in Ethiopia, and female IDPs, aged ≥18 years in Colombia.

**Results:**

The systematic review and meta-analysis identified a range of GBV experiences and estimated a 21.4 % prevalence of sexual violence (95 % CI:14.9-28.7) among conflict-affected populations. No existing screening tools for GBV in humanitarian settings were identified. Qualitative research with GBV survivors in Ethiopia and Colombia found multiple forms of GBV experienced by refugees and IDPs that occurred during conflict, in transit, and in displaced settings. Identified forms of violence were combined into seven key items on the screening tool: threats of violence, physical violence, forced sex, sexual exploitation, forced pregnancy, forced abortion, and early or forced marriage. Cognitive testing further refined the tool. Pilot testing in both sites demonstrated preliminary feasibility where 64.8 % of participants in Ethiopia and 44.9 % of participants in Colombia were identified with recent (last 12 months) cases of GBV. Implementation testing of the screening tool, conducted as a routine service in camp/district hospitals, allowed for identification of GBV cases and referrals to services. In this phase, 50.6 % of participants in Ethiopia and 63.4 % in Colombia screened positive for recent experiences of GBV. Psychometric testing demonstrated appropriate internal consistency of the tool (Cronbach’s α = 0.77) and item response theory demonstrated appropriate discrimination and difficulty of the tool.

**Conclusion:**

The ASIST-GBV screening tool has demonstrated utility and validity for use in confidential identification and referral of refugees and IDPs who experience GBV.

## Background

The humanitarian field has responded to the burden of gender-based violence (GBV) through the development and implementation of individual-level and community-targeted interventions and services. Guidelines have been established to support the development of a minimum package of services to prevent and respond to GBV in humanitarian settings, as well as to support cross-sectoral programming and response to addressing GBV [1–3]. This attention to GBV is in response to both the global burden of GBV, which is estimated to be an approximate 30 % lifetime prevalence of GBV, as well as to the increased vulnerability to GBV faced by those living in conflict and other emergency settings [4–7]. For these populations, violence may occur within the context of war or conflict, during transit and displacement, and in the camp/settlement setting. Loss of secure housing, limited economic opportunities, lack of security, and family disruption among conflict affected populations may increase vulnerability to opportunistic violence as well as intimate partner violence (IPV) [8–11] One in five female refugees or internally displaced persons (IDPs) are estimated to experience sexual violence in their lifetime; yet, even this high figure may be underestimated due to significant under-reporting of GBV [12–14]. Moreover, sexual violence is but one form of GBV experienced by women/girls. Other forms of GBV, such as physical violence and threats of violence or psychological abuse, are often experienced together, indicating that GBV among refugee and displaced populations may be even higher; however, such prevalence estimates are limited [12]. Despite gaps in understanding the burden of GBV in humanitarian settings, there is clear evidence to support programming to both prevent and respond to GBV among women and children in these settings.

In response to efforts to address GBV, many humanitarian settings now implement GBV programming, such as community outreach and education, programs to work with men and boys in the prevention of GBV, and/or women’s empowerment programs. To provide services for those who may already have experienced GBV, most settings also provide health care services, psychosocial support, and have protection available for survivors and their families [2]. Importantly, however, prevention and response programs vary in quality and availability, and factors that contribute to the variability are duration of crisis and phase of response, size of refugee/displaced population, accessibility of services, capacity of implementing and operational partners, and funding priorities [1, 9, 10]. At the minimum, humanitarian settings are expected to establish basic health, psychosocial, and protection services for survivors of GBV [1, 2]. Access to these GBV services for GBV survivors is typically based on the use of a passive approach that requires survivors or family members and communities to seek out GBV services and disclose the GBV experience. This approach requires the GBV survivor or others to: 1) *have knowledge* of available services and the rights of GBV survivors, and 2) *trust* in the confidentiality, privacy, and compassion of the person providing the service. These are challenging expectations, particularly for who have suffered multiple losses and disruption, who are forced to live in a new environment, and who may not be aware of available services in their new setting. Moreover, when providers do not initiate discussions about violence, patients may not feel empowered to initiate the discussion themselves [9, 10]. As a result, GBV continues to be under-reported, existing services under-utilized, and GBV victimization continues in humanitarian settings [12].

There are two challenges to overcoming these problems: first, in most settings there is no active approach used within clinics or other private service settings to identify and engage GBV survivors (particularly increase early engagement) in health care, protection, and psychosocial support services. Second, few interventions for GBV in humanitarian settings have been evaluated in a scientific manner [15]. Screening tools for the identification of IPV have been developed and implemented in non-conflict settings and provide an active approach to identify and increase access to services [16]. IPV screening tools have been developed and utilized in Western, high-income health care settings to facilitate identification of survivors, report cases of violence, and to improve access to health care and other services for IPV. Past trials have demonstrated effectiveness in identification of cases, engagement in care, and reductions in future violence [17]. However, screening and research on IPV tends not to address other forms of GBV, particularly those that are applicable to humanitarian settings. Moreover, despite demonstrated improvements in identification and access to care, the World Health Organizations (WHO) has not recommended universal IPV screening in health care settings due to the lack of evidence that screening reduces repeat violence in abusive relationships [18].

In response, we partnered with the United Nations High Commissioner for Refugees (UNHCR) in 2010 to develop a GBV screening tool that would strengthen early identification of survivors in order to link them to existing services that can reduce the multiple negative health and social consequences associated with GBV. With the objectives of addressing under-reporting and under-utilization of GBV-related services in humanitarian settings, we systematically developed and tested a GBV screening tool, the ‘Assessment Screen to Identify Survivors Toolkit for Gender Based Violence’ (ASIST-GBV) for use among female refugees and internally displaced women and girls. The intended outcome of the screening tool is to identify recent cases of GBV for referral to GBV services, in settings where such services are available and of adequate quality. The objective of this paper is to describe the development process of the ASIST-GBV, findings, and future use of ASIST-GBV in diverse humanitarian settings.

## Methods and Results

The development and testing of the ASIST-GBV screening tool for refugee and displaced women and girls presented three main challenges. First, was the challenge of how to narrow the broad definition of GBV to a brief, easy-to-use screening tool. The team used the UN’s definition of GBV, which states: “gender-based violence is violence that is directed against a person on the basis of gender or sex. It includes acts that inflict physical, mental or sexual harm or suffering, threats of such acts, coercion and other deprivations of liberty….” [19]. The second related challenge is the need for screening tools to be brief and easy to administer by service providers. To address the first two challenges, we used a qualitative approach and cognitive testing among GBV survivors and diverse service providers in humanitarian settings. This approach allowed us to identify contextually relevant forms of GBV; to understand the terminology used to discuss GBV with survivors so as to inform the development of the GBV screening questions; and proactively identify and address potential challenges to the implementation of the GBV screening tool by providers across various humanitarian settings.

Our third challenge was related to testing of the screening tool. As new tools are developed, they are often compared and validated against existing measures or ‘gold standards’. However, no prior tool was known to exist for GBV; thus no gold standard exists for comparison, particularly for humanitarian settings. To address this challenge, we used a composite reference standard for validation and item response theory to assess the psychometric properties of the tool.

The development and testing of the GBV screening tool was completed in four comprehensive phases: 1) systematic literature review, 2) qualitative research that included individual interviews and focus groups with GBV survivors and service providers, respectively, 3) pilot testing of the developed screening tool, and 4) 3 month implementation of the screening tool as a routine service in health care settings in camp/settlements that were different from the original sites where development and testing took place. An exploratory sequential design was used, in which the systematic review and qualitative research with survivors and service providers were intended to explore GBV in humanitarian settings (types of GBV, perpetrators, high-risk settings in camp/settlement, services and resources available to survivors) and inform the development of the tool and quantitative testing activities.

### Settings and participants

**Fig. 1:**
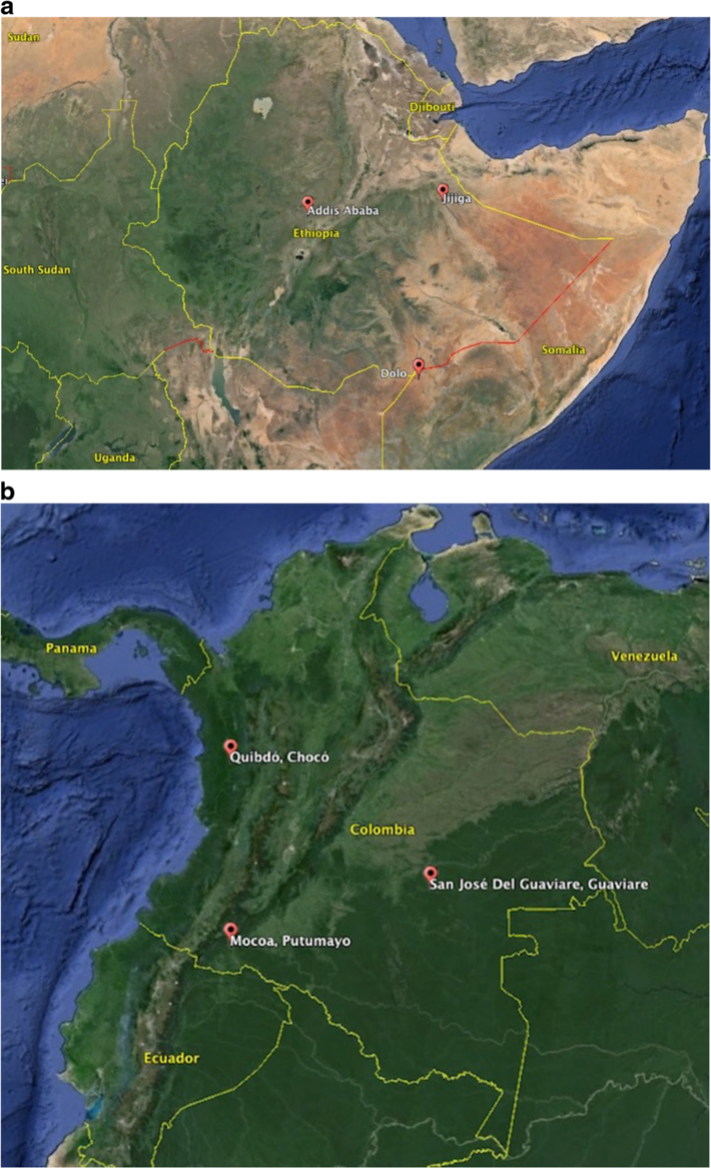
**a.** Study sites in Ethiopia. **b**. Study sites in Colombia. Image provided by U.S. Dept. of State Geographer and Landsat

Research activities were conducted among refugees living in urban and camp settings of Ethiopia and IDPs living in Colombia. Ethiopia is home to almost 730,000 refugees, as of January 2015, and predominant refugee populations originate from Somalia, Eritrea, and South Sudan [20]. Refugees from several other countries, such as the Democratic Republic of Congo, Burundi and Rwanda in the Great Lakes region of Africa also reside in Ethiopia. Colombia has one of the highest numbers of IDPs in the world and, as of December 2014, an estimated 5.8 million people were displaced within the country [21]. Most IDPs have been displaced from rural to urban areas; yet, violence in larger urban centers has led to substantial intra-urban displacement, signifying a shift in displacement modalities. Both countries were selected based on discussions with officers from UNHCR and U.S. Department of State, diversity of contexts and refugee/IDP populations, and availability of local collaborative organizations. Individual sites for all phases were based on site visits to determine appropriateness for screening (quality, availability, and confidentiality of GBV-related services) and discussions with local UNHCR and implementing partner office staff. Figure 1 displays mapped locations of where each data collection and testing phase took place in Ethiopia and Colombia.

### Human subjects protections

Gender-based violence is an inherently sensitive topic; however, few GBV interventions that are implemented for adults and adolescents in humanitarian settings are evidence-based, signifying an ethical mandate for research of new interventions [15]. To address both the lack of evidence underlying GBV interventions and the importance of participant protection, GBV-related research and programming must include procedures to protect participant confidentiality and anonymity to avoid stigmatization or further victimization by others, as well as to prevent secondary trauma during research and service provision. To this end, our research incorporated multiple procedures to protect study participants throughout all phases of research. Research practices followed institutional guidelines on human subjects research and WHO guidelines on the conduct of research on sexual violence in humanitarian settings [22]. The study was conducted in partnership with the UNHCR headquarters and offices in both countries, as well as with local implementing partners. All study methodology, protocols, consent forms and training were developed in consultation with local partners. Research protocols and instruments were reviewed locally for ethical approval by the Administration for Refugee and Returnee Affairs (ARRA), the governmental agency responsible for all refugee related concerns in Ethiopia, and the Ministry of Social Protection in Colombia. The Johns Hopkins Medical Institutes Institutional Review Board reviewed and approved all research conducted in both countries.

All data were anonymous; participants were asked not to provide their names, names of family members and friends, or other personal identifiers. Service providers who participated in qualitative research were further asked not to provide the name of the organization where they worked during data collection. All participants were consented in private using approved, translated oral consent forms. Oral consent forms avoid the collection of signatures, which would otherwise have been the only linking identifier in the study. During the consent process, all participants were informed of the study purpose and activities, voluntary nature of participation [9, 10, 23]. All GBV survivors who were included in qualitative research or pilot testing were screened and included only if they had previously received services for GBV and were determined to be mentally capable of participating in research.

Prior to pilot testing and the implementation phases, we conducted training for staff members who implemented screening or received referrals. These trainings included topics of privacy and confidentiality, conduct of screening, safety and safety planning, health needs of survivors including those aged 15–17 years, and self-care for staff supporting GBV survivors. The training and implementation process included the referral pathways established with local GBV service providers, as well as child protection programs and services. During the pilot and implementation phases, all participants were provided with information about available GBV services regardless of the outcome of their screening. Participants who screened positive for GBV were offered referrals to care. Those who agreed entered the established referral pathway by first meeting with either the GBVIMS coordinator (Ethiopia) or the hospital social worker (Colombia). This person conducted intake and then directed the survivors to appropriate services that were based on the survivors’ experiences and needs, which could include medical, reproductive health, psychosocial, and/or protection services. No identified GBV survivor was obligated to accept referral; rather, survivors were informed they could access services when they were ready to do so.

Our study team felt it was important to include young women (15–17 years) in all phases of research due to the vulnerability of young women in humanitarian settings, dearth of research on appropriate GBV interventions for them, and the need to understand how to best address this subgroup’s needs. In Ethiopia, the study received a waiver from parental consent for those under the age of 18 years, given that parents may be perpetrators of GBV or may stigmatize a child after GBV victimization [9, 10]. Young participants in Ethiopia who screened positive for GBV in pilot testing and implementation phases were offered referrals to care in the same manner as adults with additional options for referral to child protection and child-specific programs, such as programs offered by Save the Children. A waiver of parental consent was not approved in Colombia; thus, our study team decided only to include participants aged ≥18 years to avoid requesting parental consent for young participants.

### Systematic review

A systematic review was first conducted to aid in informing the development of the ASIST-GBV screening tool [14]. The primary objectives of the systematic review were to 1) identify the range of GBV experienced by female refugees and IDPs, 2) estimate the prevalence of sexual violence among female refugees and IDPs, and 3) to identify any existing screening tools that have been used for the identification of GBV in the humanitarian setting. An initial search was conducted in 2010 and subsequently repeated for publication in 2013. Following the preliminary search, the meta-analysis was restricted to a focus on sexual violence experienced complex humanitarian emergencies due to the broad definition, measurement, and interpretation of GBV in humanitarian settings [2, 19]. Multiple databases (EMBASE, CINAHL, and MEDLINE) were searched through February 2013 to identify relevant research publications. Research studies were to include the description of an evaluation of a screening tool, strategy, survey or program to identify sexual violence among female refugees, IDPs, or others in complex humanitarian settings, and estimates of sexual violence among these populations. Sexual violence was defined according to the CDC guidelines and included reported rape, non-consensual sex, molestation, sexual abuse, gang rape, marital rape, sexual violence related to exploitation, and sexual harassment, as reported by the studies’ authors [14, 24].

The systematic search returned 1,175 citations, of which 19 studies were retained after full text review. These studies were heterogeneous in participant demographic, sample size, and sampling methodology. Overall, the pooled data from a total sample of 8,398 participants from complex humanitarian settings in 14 countries provided an estimated 21.4 % prevalence of sexual violence (95 % CI: 14.9-28.7). Restriction of the analysis to studies that utilized probability-based sampling methodologies yielded a similar result of 21.0 % (95 % CI: 13.2-30.1) [14]. The estimate that one in five refugee and IDP women reported experiencing sexual violence in their lifetimes is comparable to regional estimates of the prevalence of lifetime sexual violence among women in non-conflict affected setting [5, 25]. Consequently, it is likely that the total prevalence of GBV, which encompasses multiple forms of violence, among refugees and IDPs is much higher. Substantial evidence suggests that experiences of violence are often under-reported and other forms of GBV are also highly prevalent globally and among those in humanitarian settings [5, 12, 13].

This systematic review demonstrated the burden of sexual violence (and by proxy GBV) and the need to address and respond to sexual and GBV among women in complex humanitarian settings [14]. However, no study utilized a screening tool for identification of the diverse experiences of GBV [26, 27]. Included studies reported utilizing the Harvard Trauma Questionnaire (HTQ), Abuse Assessment Screen (AAS), or other survey questionnaires to identify sexual violence. The HTQ and AAS identify traumatic experiences and intimate partner violence (IPV), respectively. Thus, despite the breadth of GBV, only limited programs have utilized screening tools for the identification of IPV or traumatic experiences in humanitarian settings [28–30].

### Qualitative research and development of the screening tool

Qualitative research was conducted to inform the development of the screening tool. These methods included in-depth interviews with refugee or IDP women who were survivors of GBV and focus group discussions conducted with service providers [9, 10]. Interviews allowed for in-depth understanding of GBV from the perspective of the survivors themselves, acceptability of asking questions about GBV in the health setting, and helped identify barriers and facilitators to accessing health services to address these needs. These qualitative methods are a valid exploratory research technique to enrich understanding and facilitate dialogue about GBV, identify potential questions to screen for GBV, understand which forms of GBV could be addressed by services in humanitarian settings, learn about a safe environment for screening, and identify barriers and challenges to implementing screening and referral procedures [31]. The in-depth interview process provides a safe and confidential environment in which the refugees/IDPs, particularly those who are GBV survivors, may feel comfortable to share their experiences. Focus group discussions, alternatively, provide informal and familiar environments in which service providers can discuss shared and distinctive experiences in provision of services, provide input on the screening tool, and offer their opinions on GBV. Together, they are an excellent method for generating domain-specific content for the development of contextually valid information and measures.

Service providers from all ranges of services (health, protection, psychosocial) were invited to participate in focus group discussions. The inclusion of service providers in qualitative research allowed for identification of other forms of GBV that may not have been reported by survivors themselves. In both settings, GBV survivors who had previously received services were identified and invited by local service providers to participate in the study. Candidate participants were preliminarily assessed for eligibility by the agencies and those who were determined not to have existing trauma were invited to the study. Only survivors who had received services and who were determined by local providers not to have pre-existing trauma were eligible. Exclusion of those who had not received services or displayed signs of pre-existing trauma was implemented to prevent potential traumatization during in-depth questioning of individuals who had not been treated for GBV. Maximum variation sampling was used to target individuals of varying age, ethnicity/origin, duration of displacement, and type of GBV.

In Ethiopia, qualitative research included 37 in-depth interviews among female refugee GBV survivors and 11 focus group discussions with health and protection providers for refugees (*N* = 77 service providers). Data were collected in March 2011 in the urban setting of Addis Ababa and three camps in the Jijiga area (Fig. 1) [9]. Addis Ababa is the capital of Ethiopia and the urban setting that houses the country offices for UNHCR, ARRA, and several implementing partner organizations. Over 1,000 refugees from Democratic Republic of Congo (DRC), Burundi, Rwanda, Sudan, Somalia and Eritrea live in Addis Ababa. Jijiga district is located in northeast Ethiopia, along the border of Somalia and Somaliland. Three refugee camps have been established in this district: Kebribeyah, Aw Barre, and Sheder, collectively accommodating over a total of 41,500 refugees, as of June 2012 [32]. The majority of the residents in the camps are refugees from Somalia.

In Colombia, qualitative research included 35 in-depth interviews among female IDPs who were GBV survivors (≥18 yrs. of age), six focus group discussions (*N* = 31 service providers) and four in-depth interviews with service providers in rural and urban settings. Data collection was conducted in San Jose del Guaviare and Quibdo in June 2012 (Fig. 1) [10]. San Jose del Guaviare is located in the department of Guaviare, which is a rural department well known for conflict and displacement. San Jose del Guaviare is a small town where displaced persons who have relocated from more remote areas comprise the majority of the population. Quibdo, in the department of Chocó, is an urban area located near the Pacific coast that receives displaced populations and also suffers intra-urban violence and displacement. Table 1 provides the demographic characteristics of qualitative participants in both countries.

**Table 1:** Demographic characteristics of qualitative research participants in Ethiopia and Colombia [9, 10]

	Ethiopia				Colombia	
Site:	Addis Ababa	Camp 1	Camp 2	Camp 3	Guaviare	Quibdo
In-depth interviews with survivors Locations and context						
Sample size:	*N* = 17	*N* = 7	*N* = 7	*N* = 6	*N* = 23	*N* = 12
Country of Origin (Ethiopia only) ^a^	Burundi, DRC, Eritrea, Somalia, Sudan	Somalia	Somalia	Somalia	N/A	N/A
Age range:	15-43 years				18-61 years	
* Total interview participants:*	*N = 37*				*N = 35*	
Provider Focus Group Discussions and Interviews						
Sample size	2 groups	2 groups	2 groups	2 groups	3 groups	3 groups, 4 interviews
Services represented:	Health services; Protection; GBV services; Humanitarian agencies				Health and psychosocial services; Humanitarian agencies; Protection and prosecutors office; Community-based women’s organizations	
* Total provider participants:*	*N = 77*				*N = 31*	

^a^N/A: participants from Colombia are internally displaced, thus originally from Colombia. Camp names excluded for protection purposes

Interview participants in Ethiopia reported rape, gang rape, abduction, imprisonment, forced witness of violence, and physical, psychological, and other sexual violence, and forced/early marriage and pregnancy. These experiences were perpetrated by armed combatants (military or rebels), unknown persons, community members, individuals with power (religious leaders, employers), intimate partners and family members and, in some cases, members of aid agencies. Experiences of violence occurred across settings including in the country of origin, host country, and during transit. Some participants were living with HIV at the time of the interview and attributed the acquired infection to past experiences of GBV [9]. Participants from Colombia reported similar forms of violence, including psychological, physical and sexual violence, sexual coercion, abduction, forced abortion, forced marriage, and forced pregnancy. Identified perpetrators and locations of GBV were wide ranging and shared similar characteristics to those reported in Ethiopia, though the contexts of the conflicts are recognizably quite different. Armed combatants, including guerrillas, paramilitary, and military and police officers, as well as strangers, community members, intimate partners and family members were identified in the list of individuals perpetrating GBV. GBV was reported to occur in the village (s)/town(s) of origin where conflict had occurred (several participants were displaced multiple times), in transit, and in the displaced setting [10].

Across the two settings, refugee and IDP women reported that they often felt that providers exhibited a lack of concern and failed to address issues of GBV. Several participants reported that health professionals rarely ask such questions and they would feel like physicians were compassionate if they began asking about or discussing GBV with their patients [9, 10]. Service providers in both areas equally reported that GBV was a problem. Physicians noted that within hospitals and clinics there were several limitations to addressing GBV experiences of survivors, which included: high demands for productivity of physicians and hospital staff as well as high patient volume. Providers overwhelmingly acknowledged the need for a GBV screening tool but emphasized that the tool should be simple, rapidly administered, and focused on forms of GBV that can be immediately addressed given the GBV services available [9, 10].

The systematic literature review and findings from the qualitative research informed the preliminary development of the ASIST-GBV screening tool. The tool was developed first with results from the Ethiopian context to include six questions that ask about experience of several GBV types (e.g. threat of violence, physical violence, sexual violence, sexual coercion, forced marriage, forced pregnancy). The screening tool was further updated from results from the qualitative research conducted in Colombia. The key revision to the screening tool following the research in Colombia was the inclusion of a question to identify forced abortion. GBV items were restricted to the last 12 months, for two reasons: 1) to identify and refer women with recent of ongoing violence and with needs for immediate services and protection, and 2) to respond to service providers’ concerns about potential burden on health services if focused on lifetime experiences of GBV [9, 10]. With respect to this last point, consideration was given to the issue that if all participants with lifetime GBV experiences were identified and referred, there would potentially be a large volume of individuals referred to GBV services who may not need immediate care. Screening for recent GBV allows for a form of triage to identify and serve most recent cases and is consistent with other commonly used IPV screening tools, which focus on the last 6 or 12 months [16]. The screening protocol included provision of information to all screening participants about available GBV services, regardless of whether they screened positive for recent GBV. This procedure aimed to raise awareness of GBV services, particularly for participants who may not yet be ready to disclose an experience of violence or who experienced violence in the past (more than 12 months prior) and who would thus screen negative as well as for others who may experience violence in the future and may need to seek services. Similarly, female genital mutilation (FGM) was excluded from the screening tool due to consideration that FGM may have been experienced by the majority of the adult female population in some contexts and, thus, screening and referral of FGM participants may potentially overwhelm camp health systems [33]. This is not intended to deny the importance of FGM, but recognizes that there are many programs to prevent and respond to FGM in humanitarian settings and attempts to avoid overwhelming the health system with FGM cases that may have already been addressed or may have no further course of treatment. The synthesis of the seven-item GBV screening questionnaire resulted from the findings from the qualitative research and systematic review and was weighed in collaboration with local partners. The seven items thus included: threats of violence, physical violence, forced sex, sexual exploitation, forced pregnancy, forced abortion, and early or forced marriage. Participants were determined to be positive for a recent experience of GBV if they responded ‘yes’ to at least one of the GBV items.

### Pilot testing phase

A testing phase was conducted after the development of the ASIST-GBV screening tool. This phase focused on assessing several features of the screening tool through cognitive testing approaches and preliminary testing with known GBV survivors and the general refugee/IDP populations.

Cognitive testing is a process that allows researchers to identify covert problems not otherwise apparent in the design and pilot testing process [34, 35]. Cognitive testing of translated items was conducted in both Ethiopia and Colombia to identify and minimize potential measurement and response errors of the screening tool. Five female refugees who were known survivors of GBV living in Addis Ababa participated in cognitive testing of the Somali, Tigrinya, and French translated versions of the screening tool and five IDP women in Colombia provided cognitive testing of the Spanish translated version of the tool. Participants in both countries were survivors who were recruited from existing GBV programs in the same process that was conducted for the qualitative phase of research [9, 10]. Participants were asked to perform several cognitive processes so as to reveal understanding of the questions and how they process and decide on response. These processes included: 1) Think-aloud: the respondents verbalized their thought processes aloud as they answer the screening questions; and 2) Paraphrase: the respondents were asked the question and then are immediately asked to repeat the question back in their own words before they have a chance to answer the question itself. The results of the cognitive testing were also used to rephrase translated questions. Cognitive testing among survivors and content validity by GBV experts further refined the English and translated versions of the screening tool prior to pilot testing.

Following revisions to the draft version of the screening tool, pilot testing of ASIST-GBV was conducted in each country. Participants in both countries were recruited from existing GBV programs or other programs for refugee/displaced women in the same manners described in previous phases. In Ethiopia, the ASIST-GBV screening tool was tested with 434 female refugees who were either self-identified female GBV survivors (*N* = 119) or women living in the refugee population in Ethiopia (*N* = 314). Preliminary testing was conducted among Somali, Congolese, Eritrean and other refugee populations in the urban refugee setting of Addis Ababa and in the three Jijiga refugee camps in June 2011 (Fig. 1). Of these, 64.8 % of the participants screened positive for at least one form of recent GBV [36]. In Colombia, pilot testing of the ASIST-GBV was tested with 69 women, including 19 self-identified GBV survivors and 50 IDP women living in San Jose del Guaviare, Colombia in October 2012 (Fig. 1). The sample size in Colombia was smaller relative to Ethiopia due to smaller population sizes in the Colombian sites and the challenges of identifying IDP participants, particularly those who are GBV survivors, in a peri-urban setting where they are much more dispersed than in a refugee camp. The numbers included in Colombia, however, were sufficient for pilot testing in this site. In Ethiopia, inclusion of a larger number allowed for testing among participants of different origins. Of Colombian women, 44.9 % screened positive for at least one form of recent GBV on the ASIST-GBV and only 15.0 % of those with a recent GBV experience had reported their case or sought services for GBV within the last 12 months [37]. Table 2 displays participant demographics, frequencies and types of GBV experiences reported during preliminary testing in each country.

**Table 2:** Demographics and recent experiences of violence, as identified by the ASIST-GBV screening tool among participants of the pilot testing phase in Ethiopia and Colombia (*N* = 503)

	Ethiopia (*N* = 434)			Colombia (*N* = 69)	
	n	Col %		n	Col %
Site					
Camp	86	19.8	Guaviare	69	100.0
Addis Ababa	348	80.2			
Median age (range)	29	(15–90)		39	(19–78)
Median years in camp/displaced setting (range)	4	(0–21)		8	(0–28)
Marital status					
Married/Living together as married	213	49.3		32	46.4
Separated/Divorced/Widowed	145	33.6		25	36.2
Never married	74	17.1		12	17.4
Country of origin / Ethnicity					
Burundi	7	1.6	Afro-Colombiana	4	6.2
DRC	18	4.2	Indigena	2	3.1
Eritrea	32	7.4	Mestizo/Blanco	56	86.2
Kenya	2	0.5	Other	3	4.6
Somalia	371	85.7			
Uganda	1	0.2			
Other	2	0.5			
Self-reported history of GBV (lifetime)					
No	314	72.5		50	72.5
Yes	119	27.5		19	27.5
Recent GBV (ASIST-GBV items)					
Threatened/insulted	245	56.6		18	26.1
Physically hurt	187	43.2		13	18.8
Forced sex	110	25.5		9	13.0
Sexual exploitation	49	11.3		7	10.1
Forced pregnancy	78	18.1		2	2.9
Forced abortion^a^	NA	NA		1	1.4
Forced marriage	41	9.5		2	2.9
Any type of GBV	282	64.8		31	44.9
Accepted a referral (if ASIST-GBV is positive)	157	55.9		27	90.0

^a^Screening item about forced abortion not included in Ethiopian instrument

Developed screening tools are often compared to gold standard measures to assess validity of the tool. Because no previous tool has been developed to identify GBV in humanitarian settings and because other screening tools for IPV or trauma would likely fail to identify some forms of GBV, we focused on the use of item response theory to further assess the psychometric properties of ASIST-GBV among a larger sample of women during the implementation phase.

### Implementation phase with psychometric testing

The final phase of the development and testing of the ASIST-GBV screening tool was dedicated to assessing the performance of the tool when implemented by humanitarian agencies among a naive population, a population who had not yet been exposed to the screening tool during the previous phases. Screening was conducted in Ethiopia by local implementing partners, the Partnership for Pastoralists Development Agency (PAPDA), which provided GBV psychosocial services and managed the Gender-based Violence Information Management System (GBV-IMS; www.gbvims.com) in the Bokolomayo refugee camp in the Dolo Ado area (Fig. 1). Screening was implemented for three months, between June and August 2012 and was conducted in the ARRA camp health clinic among women attending clinical, reproductive or antenatal, and/or their children’s health visits. In this setting, screening was promoted as a routine service offered to all women attending the clinic, which allowed for women to be screened without raising community suspicion that select women were being targeted for screening. Prior to screening, participants underwent a verbal informed consent process in which they were informed of the content of the questions, voluntary nature of participation, and that no services would be affected if they were to decline participation [23]. Participants were privately consented and screened by Somali speaking social workers. Participants who screened positive for GBV were offered referral to established, appropriate services, including medical, reproductive health, psychosocial, and protection services, based on individual GBV experiences and needs. Screening and identification of survivors with recent GBV experiences led to subsequent referral and enrollment of the survivors’ information into the GBV-IMS, for those who provided additional consent. The GBVIMS is monitoring system developed for collection of GBV data and sharing of information in humanitarian settings. During implementation testing, 487 were screened; 50.6 % reported some form of GBV (last 12 months) and were offered referrals. Of these, 43.8 % accepted the referral at the time it was offered.

As in Ethiopia, the implementation phase testing was conducted by nurses hired and trained by the study team within a district hospital in Mocoa, Colombia (Fig. 1). A total of 511 women from the general IDP population were enrolled and screened from February to May 2013. Participants were recruited from the adult population of women attending health visits, antenatal/reproductive health visits in the clinic. Here, too, screening was promoted as a standard hospital service offered to all women aged 18 years and older. Participants were consented and screened in a private room and offered direct referral to the hospital’s social worker that managed all GBV services and external referrals. Among participants, 63.4 % screened positive for at least one form of GBV, as determined by the ASIST-GBV screening tool and, of these, 74.2 % accepted the referral at time it was offered. Table 3 presents the demographic distribution and results of the ASIST-GBV for both sites. It is worth noting that there is the possibility that some participants were double counted in these estimates (Table 3), given that to protect participant confidentiality, we did not collect identifiers to determine previous participation. Any participant who reported being previously screened were given the option to be screened again or decline. Screening a second time is not a concern because repeated screening allows for identification of new cases of GBV that arose since last screening and referral for care. Also, estimating the prevalence of GBV, which would be concerned with double counting, is neither the aim of the study nor of screening. Across both sites, the social workers and nurses who administered the screening tool commented on the benefit of promoting the tool as a standard practice offered by the hospital, in terms of increasing comfort with the screening and reducing concerns about targeting of the screening or subsequent social stigma.

**Table 3:** Demographics and recent experiences of violence, as identified by the ASIST-GBV screening tool among participants of the implementation phase in Ethiopia and Colombia (*N* = 998)

	Country				
	Ethiopia (*N* = 487)			Colombia (*N* = 511)	
	n	Col %		n	Col %
Demographics					
Camp/Town where Implementation Phase was conducted					
Bokolomayo	483	100.0	Mocoa	508	100.0
Median age (range)	29	(15–81)		29	(18–62)
Refugee Country of Origin			Colombian Ethnicity		
Somalia	480	100.0	Mestizo/Blanco	389	76.9
			Afro-Colombiana	43	8.5
			Indigena	72	14.2
			Raizal de Archipelago	2	0.4
			Other	1	0.2
Median years in camp/displaced setting (range)	2	(0–20)		7	(2–15)
Marital Status					
Married/Living together	401	84.1		291	57.4
Formerly married	39	8.2		60	11.8
Single	37	7.8		156	30.8
Education (completed)					
Never	249	56.7		13	2.6
Pre-school or primary	136	31.0		198	39.9
Secondary	51	11.6		244	49.2
Technical	0	0.0		41	8.3
University or higher	3	0.7		0	0.0
Recent GBV (ASIST-GBV items)					
Threatened/insulted	172	35.7		210	41.5
Physically hurt	224	46.6		119	23.5
Forced sex	98	20.4		182	36.0
Sexual exploitation	133	27.7		102	20.2
Forced pregnancy	76	15.8		10	2.0
Forced abortion	NA	NA		8	1.6
Forced marriage	95	19.9		21	4.2
Any type of GBV	244	50.6		319	63.4
Accepted a referral (if ASIST-GBV is positive)	105	43.8		236	74.2

N/A: Screening item about forced abortion not included in Ethiopian instrument; some participants may have been screened more than once

Data from the implementation phase in both sites were combined and utilized for additional psychometric analysis (the item related to forced abortion was excluded from this analysis due to the fact that it was not included in the Ethiopian version of the screening tool). Psychometric methods included the use of Cronbach’s α coefficient, factor analysis, and item response theory (IRT) [23]. Cronbach’s α coefficient enables the estimation of the internal consistency of the construct and was high at 0.77. Exploratory factor analysis using principle components was conducted to assess if the 6-items included in the screening tool were unidimensional and found that all items loaded onto a single factor (factor loadings: threat of violence 0.51, physical violence 0.65, forced sex 0.58, survival sex 0.69, forced pregnancy 0.63, and forced marriage 0.57). IRT examines the relationship between a participant’s position on a latent trait (in this case, the degree of GBV) and the probability that they endorse different items on the GBV screening tool. In this case, IRT offers better utility for estimating the psychometric properties of a screening tool than classical test theory approaches [38]. IRT allows for measurement of difficulty and discrimination across the items and demonstrated low difficulty among items such as threats of violence (0.690) to higher difficulty among items such as forced marriage (3.51) and forced pregnancy (6.33) with appropriate discrimination to identify different forms of GBV. Differential item functioning (DIF) was tested across countries and demonstrated that characteristics of the GBV screener are the same for the two countries [23].

## Discussion

The ASIST-GBV screening tool has undergone an extensive process of development and testing and successfully identifies threats of violence, physical and sexual violence, sexual exploitation, forced pregnancy and abortion, and forced/early marriage among women in humanitarian settings. Our research has demonstrated that the screening tool has strong psychometric properties to identify and discriminate across the range of GBV types and increases confidential identification and referral of GBV cases. The tool can be rapidly administered and confidentially used in low-resourced settings by trained providers and has demonstrated feasibility for implementation in settings where GBV services of adequate quality already exist. Trained, entry level staff, nurses, and social workers can implement the screening tool and reduce effort from physicians who may be present in lower numbers than other staff members and who may have limited time for screening. Since the development and testing, the screening tool has been further used with female refugees in Uganda, Lebanon, and Kenya; these data are currently being evaluated and may lend to cross-cultural validation to the tool for use in other settings outside of the development and testing phases settings and apart from populations among whom it was initially developed. While some IPV screening tools are now being used in African settings, the use of the ASIST-GBV screening tool would additionally identify women who have experienced non-partner violence [39]. It is important to note, however, that the ASIST-GBV screening tool should be used only in settings where health, protection, and psychosocial service are available for referral and are of adequate quality and confidentiality. Moreover, the referral pathway should be clearly defined and all screening staff trained on the referral pathway, prior to implementation of screening. Such pre-requisites and staff training for implementation of screening are described in the ASIST-GBV manual for use [40]. Where such services are not available or are of inadequate quality and confidentiality, it is not appropriate to implement screening until services are available and meet humanitarian standards.

Overall, the tool supports existing GBV programs through early identification and referral to needed health, safety and protection services, and has several features that provide additional benefits and sustainability. The requirement of a referral system for survivors identified through screening encourages mapping and increased coordination across the diverse sectors (e.g. protection, health, water, logistics) working in the settings [1, 2]. Additionally, many humanitarian settings also have reporting mechanisms and monitoring systems, such as the GBV-IMS, to which the screening tool can be linked. The use of the ASIST-GBV screening tool does not duplicate these mechanisms but supports them, as it does services, through identification and direct linkage to the referral pathway. The ASIST-GBV identifies similar types of violence to those identified by the GBV-IMS, allowing for information obtained via screening to be translated to the GBV-IMS, avoiding repeat questions and potential retraumatization of survivors by requiring them to answer the questions multiple times. Regular use of the ASIST-GBV screening tool and questions about GBV experiences in a community may ultimately serve to change social norms and awareness related to GBV and inform the population that confidential GBV services exist, should they need them in the future [9, 10, 41].

The tool may have applicability outside of humanitarian settings. Given the dispersal of IDPs among the general population in Colombia, feedback from hospital staff where the implementation phase was conducted suggested that the tool was also useful among the general population of women who experience GBV [42]. We have since expanded implementation research among the general population in Somalia. Given the global prevalence of non-partner violence, the ASIST-GBV screening tool may be relevant for a range of countries beyond the humanitarian response [4, 25].

Despite the amount of research that went into the development and testing of the screening tool, several questions remain. First, despite the high uptake of screening and generally positive feedback, we do not have high quality measures on the acceptability of the tool among end-users, such as the target refugee/IDP population and the service providers who utilized the screening tool. Such research is currently underway and could further improve upon the content of the screening tool or methods in which it is implemented in the clinic setting. Additionally, further research is needed on the safety of screening and final outcomes of referral. Our research was limited to cross-sectional studies and while no adverse events were reported, it is important to implement longitudinal research that can determine if there are increases or reductions in violence following screening and to assess the uptake of referrals that are provided to survivors. Understanding the uptake of referrals is also important for GBV programming, where assessments of potential barriers or inefficiencies in the referral process provide a quality improvement function. We do not know the effectiveness of the screening tool in identifying GBV and providing referrals, relative to the current standard, passive approach. Addressing these remaining questions may provide an opportunity for widespread use of a sustainable, evidence-based screening tool that supports current GBV services and surveillance systems in humanitarian settings.

Finally, it is important to note that the tool described here was developed only for use among women and girls. We have conducted similar work to develop a screening tool for use among male refugees, recognizing that men and women have different experiences of GBV and may need to be asked about experiences of violence in different manners [43]. Moreover, available services of adequate quality to address GBV among women may not be adequate for responding to violence among men and boys, particularly traumatic sexual violence; thus, capacity for responding to GBV or sexual violence against men may need to be improved prior to screening male refugees/IDPs even where GBV services for women may already exist.

## Conclusions

The ASIST-GBV screening tool has undergone extensive evidence-based development and testing among refugees and IDPs in Ethiopia and Colombia, respectively. It has been developed for use in settings where services to address and respond to GBV exist and are of adequate quality. In these contexts, the ASIST-GBV screening tool captures a range of recent GBV experiences for confidential identification and referral of survivors for care and may be an efficient method to improve access to existing, GBV-related services and monitoring in humanitarian settings.

## Abbreviations

AAS: Abuse Assessment Screen
ARRA: Administration for Refugee and Returnee Affairs
ASIST-GBV: Assessment Screen to Identify Survivors Toolkit for Gender - Based Violence
CDC: U.S. Centers for Disease Control
GBV: Gender-based Violence
HTQ: Harvard Trauma Questionnaire
IDP: Internally Displaced Persons
IPV: Intimate Partner Violence
UNHCR: United Nations High Commissioner for Refugees
